# Domestic Correspondence

**Published:** 1889-07

**Authors:** 


### Domestic Correspondence.

To the Editor:

At the semi-annual meeting of the Massachusetts Dental Society, held in Boston, June 5 to 7, 1889, the following resolutions of respect were passed upon the death of Dr. F. Searle, of Springfield:

"*Resolved*, That the Massachusetts Dental Society, in the death of Dr. F. Searle, has lost an old and valued member, whose influence was always exerted to elevate the profession and increase its usefulness. The Society recognizes the great value of his services to the community in which he lived for so many years, and also his generous contributions to the theory and practice of our art, the result of his thoughtful study and investigations. We mourn the loss of a friend and father.

"*Resolved*, That a copy of these resolutions be sent to the family, the Springfield papers, and the dental journals.

(Signed) "L. D. Shepard,
"R. R. Andrews,
"G. A. Maxfield,
"*Committee on Resolutions.*"

---

At the semi-annual meeting of the Massachusetts Dental Society, held in Boston, June 5 to 7, 1889, the following resolutions of respect were passed upon the death of Dr. Leon Rideout, of Lynn, Massachusetts:

"Whereas, The all-wise Father has removed from our ranks our friend and associate Dr. Leon Rideout, of Lynn, Massachusetts:

"*Resolved*, That, in the death of Dr. Rideout, this Society has lost one of its most valued members, a man of excellent judgment and skill in his profession, interested in every movement tending towards its advancement, one who was highly esteemed in the community in which he lived and eminently successful in his calling. Genial in disposition, beloved by all who knew him, his presence will be missed from among us.

(Signed) "S. G. Stevens,
"A. W. Howland,
"S. F. Ham,
"*Committee on Resolutions.*

"Edgar O. Kinsman, *Secretary of the Society.*

"Cambridge, Mass., June, 1889."

To the Editor:

I thank Dr. Rhein for his sharp criticism of my queries in regard to the operation performed for Dr. Andrews by Dr. Ottolengui. The questions were asked to elicit more light.

Dr. Rhein will permit me, however, to observe that I do not admit, as he says I do, that abraded surfaces on the cutting edges of "incisors" present a most inviting field for "bacteria to promote caries."

Any smooth surface that can be kept free from deposits on every side is practically free from caries so long as it is kept clean.

E. A. Bogue.

New York, June 11, 1889.

---

To the Editor:

At the annual meeting of the New York State Dental Society, held in the city of Albany on May 8 and 9, 1889, the following-named were elected officers for the ensuing year: J. Edward Line, President; W. W. Walker, Vice-President; F. T. Van Woert, Recording Secretary; H. G. Mirick, Treasurer; C. L. Curtis, Correspondent. Board of Censors: William Carr, William Jarvie, S. D. French, W. H. Colgrove, S. B. Palmer, A. M. Holmes, Frank French, and A. P. Southwick.

F. T. Van Woert.

---

To the Editor:

Would the average practitioner be justified in the extraction of a superior temporary cuspid, at the age of nine and a half years, for the purpose of making space in the regulation of "axioverted central incisors," the lateral being subsequently pushed towards the space vacated by the extracted cuspid? (See *Cosmos*, p. 231.)

Is it not very probable that the permanent cuspid will be crowded out of the arch?

Why not cut away the crown of the temporary tooth sufficiently to gain the needed space?

